# Rapid-onset dystonia-parkinsonism is associated with reduced cerebral blood flow without gray matter changes

**DOI:** 10.3389/fneur.2023.1116723

**Published:** 2023-01-26

**Authors:** Christopher T. Whitlow, Kyle M. Atcheson, Beverly M. Snively, Jared F. Cook, Jeongchul Kim, Ihtsham U. Haq, Kathleen J. Sweadner, Laurie J. Ozelius, Allison Brashear

**Affiliations:** ^1^Section of Neuroradiology, Department of Radiology, Wake Forest University School of Medicine, Winston-Salem, NC, United States; ^2^Department of Biomedical Engineering, Wake Forest University School of Medicine, Winston-Salem, NC, United States; ^3^Clinical and Translational Science Institute, Wake Forest University School of Medicine, Winston-Salem, NC, United States; ^4^Department of Biostatistics and Data Science, Wake Forest University School of Medicine, Winston-Salem, NC, United States; ^5^Department of Psychiatry and Behavioral Medicine, Wake Forest University School of Medicine, Winston-Salem, NC, United States; ^6^Department of Neurology, University of Miami, Miami, FL, United States; ^7^Department of Neurosurgery, Massachusetts General Hospital, Boston, MA, United States; ^8^Department of Neurology, Massachusetts General Hospital, Boston, MA, United States; ^9^Department of Neurology, Jacobs School of Medicine and Biomedical Sciences, University at Buffalo, Buffalo, NY, United States

**Keywords:** voxel-based morphometry, rapid-onset dystonia-parkinsonism (RDP), *ATP1A3*, cerebral blood flow (CBF), arterial spin labeling

## Abstract

**Purpose:**

Previous research showed discrete neuropathological changes associated with rapid-onset dystonia-parkinsonism (RDP) in brains from patients with an *ATP1A3* variant, specifically in areas that mediate motor function. The purpose of this study was to determine if magnetic resonance imaging methodologies could identify differences between RDP patients and variant-negative controls in areas of the brain that mediate motor function in order to provide biomarkers for future treatment or prevention trials.

**Methods:**

Magnetic resonance imaging voxel-based morphometry and arterial spin labeling were used to measure gray matter volume and cerebral blood flow, respectively, in cortical motor areas, basal ganglia, thalamus, and cerebellum, in RDP patients with *ATP1A3* variants (*n* = 19; mean age = 37 ± 14 years; 47% female) and variant-negative healthy controls (*n* = 11; mean age = 34 ± 19 years; 36% female).

**Results:**

We report age and sex-adjusted between group differences, with decreased cerebral blood flow among patients with *ATP1A3* variants compared to variant-negative controls in the thalamus (*p* = 0.005, Bonferroni alpha level < 0.007 adjusted for regions). There were no statistically significant between-group differences for measures of gray matter volume.

**Conclusions:**

There is reduced cerebral blood flow within brain regions in patients with *ATP1A3* variants within the thalamus. Additionally, the lack of corresponding gray matter volume differences may suggest an underlying functional etiology rather than structural abnormality.

## 1. Introduction

Rapid-onset dystonia parkinsonism (RDP) is a rare autosomal dominant form of dystonia which has been associated with variants in the *ATP1A3* gene (1–3). These patients classically present with onset of symptoms over the span of hours to days that eventually progresses to a body-wide dystonia with prominent bulbar symptoms, and the onset is often associated with environmental stressors such as childbirth, extreme exertion, fever/infection, psychological stress or binge alcohol drinking (1, 2, 4). Furthermore, the phenotype can vary significantly amongst individuals with the same *ATP1A3* variant and in both familial and *de novo* cases (2).

Identifying variants within the *ATP1A3* gene as the underlying etiology for these patients has allowed for improved diagnosis of RDP and for identifying atypical presentations that expand the spectrum of *ATP1A3* disease (5). Given the challenge of clinically diagnosing and tracking disease symptomatology in RDP patients, noninvasive imaging correlates of disease presence or symptom severity are needed to guide clinical management and define outcomes for potential treatment trials. The diagnosis of RDP is currently based on a positive variant in the gene and a history of motor symptom onset after 18 months of age, with imaging not currently used for diagnostic purposes (2). RDP patients seldom show structural changes with standard brain imaging (6–11). However, advanced neuroimaging techniques have been used as investigative tools in dystonia. For instance, hypometabolism was detected in *ATP1A3* alternating hemiplegia of childhood (AHC) patients by SPECT or FDG-PET prior to the availability of genetic confirmation (10, 12). Additionally, magnetic resonance imaging (MRI) diffusion tensor imaging has revealed dystonia-associated white matter tract abnormalities involving components of the cortico-striato-pallidothalamocortical (CSPTC) and cerebello-thalamo-cortical (CbTC) pathways in patients with *TOR1A* mutations (13–17). In the work described in this manuscript, we have combined high-resolution structural and functional MRI methods and more advanced analysis techniques, such as voxel-based morphometry (VBM) of gray matter (GM) volume and measures of cerebral blood flow (CBF) using arterial spin labeling (ASL).

The purpose of this investigation was to determine if quantitative structural and functional MRI methodologies identify differences between patients with *ATP1A3* variants and variant-negative controls in areas of the brain that mediate motor function. We hypothesized that VBM analysis could be used to infer cell loss that may occur as a downstream consequence of pump inactivation or protein misfolding (18). Previous studies have also shown that CBF and oxygen consumption are positively correlated in normal brain in areas that are stimulated, with the proposition that alterations in energy metabolism may be detectable by measures of CBF (19–21). Therefore, CBF imaging modalities could be germane in evaluating RDP patients given *ATP1A3* encodes the neuron-specific Na, K-ATPase, which consumes up to 50% of the energy used in the brain (3, 22). The brain regions of interest were chosen based on *ATP1A3* neuropathology (23) and neuroanatomical sources of the complex motor loops (CSPTC and CbTC pathways) that mediate motor dysfunction in dystonia (13–17). This approach is relevant for further evaluating these movement disorders with the hope of identifying specific regions of the brain involved in the motor manifestations, as an imaging biomarker could help track treatment response to medication and device interventions as well as help track the natural history of the disease.

## 2. Methods

### 2.1. Subjects

All participants signed an informed consent form in accordance with the Declaration of Helsinki and approved by the Institutional Review Board (IRB00007686) before contributing a blood or saliva sample for DNA screen for *ATP1A3* variants by direct sequencing as described previously (1). Based on the current consensus on RDP (24), we adopted minimal criteria to define our cohort. Inclusion criteria were having an *ATP1A3* variant and motor symptom onset after 18 months of age (2) (cases) or being a healthy, variant-negative family member (controls) to a participating patient for frequency matching (cases to controls). Genetically-related family member healthy controls were used specifically to better match cases and controls in the presence of intra-familial correlation in outcome measures. In total, we studied 19 RDP patients from 12 families (1–5 patients in each family) and 11 variant-negative family members in seven of the families (1–4 controls from each family). Healthy controls of the same sex and similar ages as patients were preferentially recruited to maintain similar distributions by sex and age (patients: mean age ± SD = 37 ± 14 years, 47% female; controls: mean age ± SD = 34 ± 19 years, 36% female) (Table 1). The full clinical cohort has been characterized previously (2). No participants with *ATP1A3* variants were receiving specific pharmacotherapy for RDP at the time of clinical assessment or neuroimaging.

**Table 1:** Demographics by variant status: count (%), mean ± SD, and median [interquartile range].

**Characteristic**	**Overall**	**Variant-positive**	**Variant-negative**	***P* value**
	**(*n =* 30)**	**(*n =* 19)**	**(*n =* 11)**	
Female	13 (43%)	9 (47%)	4 (36%)	0.71
Age at examination, years	36 ± 16	37 ± 14	34 ± 19	0.28
	34 [25.25-47.5]	39 [28.5-48]	31 [18-42]	
BFMDRS	26 ± 28	40 ± 26	0.68 ± 1.3	< 0.001^*^
	19 [0.25- 38]	35.5 [22.5-50.5]	0 [0-0.50]	
UPDRS (*n =* 17, 10)	26 ± 23	39 ± 18	3.1 ± 2.1	< 0.001^*^
	22 [4.5-41.5]	38 [32-49]	3.5 [2-4.75]	
Onset age, years, (*n =* 18)	-	21 ± 1	-	-
		21 [15.5-23]		
< 18 years	-	6 (33%)	-	-
18–25	-	8 (44%)	-	-
26-39	-	3 (17%)	-	-
>40	-	1 (5.6%)	-	-
Duration of RDP, years (*n =* 18)	-	18 ± 13	-	-
		14.5 [7.75-29.5]		

BFMDRS, Burke-Fahn-Marsden Dystonia Rating Scale, total score; UPDRS, Unified Parkinson's Disease Rating Scale, motor score.

Results are based on n = 19 variant-positive and n = 11 variant-negative, unless otherwise noted.

P values are for comparison between variant-positive and negative groups, based on Fisher's exact and Wilcoxon rank sum tests. P values ≤ 0.05 (^*^) are considered statistically significant.

### 2.2. Medical history/movement disorder assessment

Standardized videotaped motor assessments were administered by a neurologist with expertise in dystonia (AB and IH), using the Unified Parkinson's Disease Rating Scale (UPDRS) and Burke-Fahn-Marsden Dystonia Rating Scale (BFMDRS). The BFMDRS was used to assess severity and frequency of dystonia in nine body areas including eyes, mouth, speech or swallowing, neck, right and left arms, trunk, and right and left legs (25). A standardized questionnaire was also administered to establish subjects' medical history, including family history, age and site of onset, severity of symptoms, triggers, second events of symptom onset, and self-reported education history. For the UPDRS and BFMDRS, the total motor subscale score and total score were used, respectively.

### 2.3. MRI data acquisition

Imaging data, including T1-weighted anatomic and ASL functional images were acquired using a 3T Siemens Skyra MRI scanner with a 32-channel head coil (Siemens, Erlangen, Germany) *via* standard techniques. T1-weighted anatomic images were obtained using a 3D volumetric MP-RAGE sequence (TR = 2,300 msec; TE = 2.98 msec; TI = 900 msec; FA = 9 degrees; 192 slices, voxel dimension = 1 mm isotropic). FLAIR images were acquired using a 3D SPACE inversion recovery sequence (TR = 6,000 ms; TE = 285 ms; TI = 2,200 ms; FA = 120 degrees; 160 slices, voxel dimensions = 1.1 x 1.1 x 1 mm). Whole-brain resting baseline CBF was acquired with a pseudocontinuous ASL (PCASL) sequence (26). During this functional MRI scan, participants were instructed to lie still and stare at cross-hairs that they could see in the scanner. PCASL scan parameters include: tagging duratio*n* = 1.8 s, post-labeling delay = 1.2 s, TR = 4 s, repetitions = 48, FOV = 22 x 22 cm, image resolution of 64 x 64 x 26 voxels @ 3.4375 x 3.4375 x 6 mm, with a single shot EPI acquisition, 26 slices, and acquisition time = 3 min and 28 seconds. The majority of subjects did not receive medication for purposes of anxiolysis/sedation at time of MRI. Two RDP patients received low doze benzodiazepines (0.25 mg alprazolam in one patient and 2 mg of diazepam in the other) for the purpose of imaging acquistion. Patients continued to take their typical medications as per their usual regimen on day of scan.

### 2.4. MRI data processing

Quantitative structural volumetric data processing included segmentation into GM, white matter and cerebrospinal fluid, normalization to Montreal Neurological Institute/International Consortium for Brain Mapping (MNI/ICBM) standard space, and modulation with the Jacobian determinants of the warping procedure to generate volumetric tissue maps using the DARTEL high-dimensional warping and the Statistical Parametric Mapping 8 new segment procedure, as implemented in the VBM8 toolbox (Wellcome Department of Imaging Neuroscience, University College London, UK) (27–29). Region of interest-based measures were generated using the Automated Anatomical Labeling (AAL) atlas (30). GM volumes were adjusted for total intracranial volume (comprised of GM + white matter + cerebrospinal fluid). Quantitative functional CBF data processing was performed *via* in-house Matlab algorithms and SPM12, and included data cleaning (removal of individual images with noise spikes or motion artifact), realignment (separately for label and control images), resampling to native T1 image resolution of 256 x 256 x 192 @ 1 mm isometric, spatial smoothing with an 8 mm full-width at half-maximum 3D isotropic Gaussian kernel, and calculation of mean CBF into the physiological unit (ml/100g tissue/min) (31, 32). Region of interest (ROI) metrics (AAL atlas) were then generated using the harmonized structural transformations from linear/nonlinear registrations.

### 2.5. Statistical analysis

Differences between variant-positive and variant-negative participants were tested at alpha level = 0.05 using Fisher's exact and Wilcoxon rank sum tests. Multiple linear regression was conducted for the MRI measures among all study participants, with variant status as the explanatory variable of interest, adjusted for sex and age at time of MRI. The impact of multiple comparisons was reduced by: (1) limiting the number of regions, (2) summing across left and right regions, and (3) summing across cerebellar substructures. Regions included for analyses were limited to those determined *a priori*, and several AAL atlas substructures were summed as follows: All right and left substructures were summed (e.g., left thalamus + right thalamus = target region “thalamus”), and all AAL atlas cerebellar substructures (cerebellum I-IX) were summed to produce the target region “cerebellum.” This approach resulted in the final seven target ROIs used for analyses: pallidum, cerebellum, precentral gyrus, supplementary motor cortex, caudate nucleus, putamen, and thalamus. The GM volumes were divided by intracranial volumes prior to analyses. As part of the analyses, residuals were examined to identify if data transformation would be useful. Also, potentially influential observations were identified and their impact examined. This included observations from two variant-negative participants with ages 8 and 70 years, which were outside the range of ages (14–64 years) among the variant-positive patients. As in the evaluation of variant effects, multiple linear regression was conducted for the MRI measures to evaluate potential associations with the UPDRS and BFMDRS motor measures among the variant-positive RDP patients, adjusted for sex and age. Regression results are expressed as coefficients and 95% confidence intervals; *P* ≤ 0.05/7 = 0.007 are considered statistically significant, adjusting for the seven regions of interest using the Bonferroni approach.

## 3. Results

The demographics of the subjects are given in Table 1. Results of the age- and sex-adjusted models for analyses are presented as the regression coefficients for GM volume and CBF MRI measures among all study subjects with variant status as the explanatory variable of interest, along with the calculated confidence intervals (Table 2). There were statistically significant between group differences in BFMDRS and UPDRS (*p* ≤ 0.001) (Table 1), which is typical of RDP. Thus, a second hypothesis was tested that a relationship would be found between MRI measures and motor performance (Table 3). Though nonmotor/cognitive changes are also commonly seen in RDP (2, 33), we focused on motor outcomes in this study.

**Table 2:** Effects of variant status: multiple linear regression coefficients (95% confidence intervals) for MRI measures among all study subjects—variant status as explanatory variable of interest.

	**Gray matter volume**		**Cerebral blood flow**	
**Region of interest**	**Estimate [95% CI]**	***P* value**	**Estimate [95% CI]**	***P* value**
Pallidum	−0.078 [−0.23, 0.071]	0.29	−11.2 [-22.2,−0.3]	0.045
Cerebellum	1.4 [−3.9, 6.7]	0.59	−22.2 [-39.7,−4.6]	0.015
Precentral gyrus	0.35 [−1.8, 2.4]	0.74	−22.5 [-43.9,−1.1]	0.040
Supplementary motor cortex	−0.43 [−1.8, 0.92]	0.52	−23.6 [-44.5,−2.7]	0.029
Caudate nucleus	0.59 [−0.36, 1.6]	0.21	−17.9 [-34.2,−1.7]	0.032
Putamen	−0.12 [−1.1, 0.82]	0.79	−15.4 [-32.4, 1.5]	0.073
Thalamus	−0.00065 [−0.812, 0.811]	0.99	−28.2 [-47.1,−9.3]	0.0051^*^

Results are based on complete data, n = 30 (19 variant-positive, 11 variant-negative) and are adjusted for covariates age and sex. Bonferroni-corrected P values ≤ 0.007 (^*^) are considered statistically significant.

**Table 3:** MRI and motor measure association estimates: multiple linear regression coefficients (95% confidence intervals) for MRI measures among variant-positive participants—motor measures as explanatory variables of interest.

	**Gray matter volume**		**Cerebral blood flow**	

**Region of interest**	**BFMDRS coefficient** **[95% CI]** **(*****p*** **value)**	**UPDRS coefficient** **[95% CI]** **(*****p*** **value)**	**BFMDRS coefficient** **[95% CI]** **(*****p*** **value)**	**UPDRS coefficient** **[95% CI]** **(*****p*** **value)**
Pallidum	0.0013 [−0.0017, 0.0044] 0.37	0.00051 [−0.0037, 0.0048] 0.80	−0.019 [−0.28, 0.24] 0.87	−0.026 [−0.42, 0.37] 0.89
Cerebellum	0.048 [−0.11, 0.21] 0.53	0.063 [−0.19, 0.32] 0.60	0.050 [−0.37, 0.47] 0.80	0.027 [−0.56, 0.61] 0.92
Precentral gyrus	0.0070 [−0.040, 0.054] 0.76	0.025 [−0.046, 0.097] 0.46	0.10 [−0.33, 0.54] 0.63	0.13 [−0.47, 0.73] 0.64
Supplementary motor cortex	0.0012 [−0.026, 0.029] 0.93	−0.0012 [−0.045, 0.042] 0.95	0.11 [−0.29, 0.51] 0.55	0.13 [−0.33, 0.59] 0.55
Caudate nucleus	0.014 [−0.011, 0.038] 0.25	0.014 [−0.0085, 0.037] 0.20	0.14 [−0.23, 0.50] 0.44	0.16 [−0.40, 0.72] 0.55
Putamen	−0.00096 [−0.024, 0.022] 0.93	−0.011 [−0.042, 0.020] 0.47	0.065 [−0.30, 0.43] 0.71	−0.035 [−0.59, 0.52] 0.89
Thalamus	0.00020 [−0.0179, 0.0183] 0.98	−0.0033 [−0.033, 0.026] 0.81	−0.21 [−0.72, 0.29] 0.39	−0.36 [−1.1, 0.38] 0.32

BFMDRS, Burke-Fahn-Marsden Dystonia Rating Scale total score UPDRS, Unified Parkinson's Disease Rating Scale motor subscale.

Results are from separate models for each motor measure, n = 19 subjects with BFMDRS and n = 17 with UPDRS, and are adjusted for covariates age and sex.

There was a statistically significant (*p* ≤ 0.007) age and sex-adjusted between group difference in CBF, with lower rates of CBF among patients with RDP compared to variant-negative controls in the thalamus (Table 2). Multiple additional areas demonstrated decreases in CBF that were not statistically significant, including the pallidum (*p* = 0.045), cerebellum (*p* = 0.015), precentral gyrus (*p* = 0.040), supplementary motor cortex (*p* = 0.029), and caudate nucleus (*p* = 0.032) (Table 2). For measures of GM volume, there were no statistically significant between group differences for ROIs (all *p* ≥ 0.007) (Table 2). In addition, no statistically significant (all *p* ≥ 0.007) relationships were detected between motor function (UPDRS and BFMDRS), MRI measures of GM volume or CBF (Table 3), or disease duration (results not shown).

Although age was used as a covariate for these analyses, it is possible that patient age may still play a role in the functional brain MRI findings described, as the known decreased CBF with increasing age may be non-linear across the large age range in this study. Additional analyses (results not shown) removing the youngest and oldest individuals diminished the between group differences in CBF somewhat but did not alter the direction of between group differences in CBF, with the lower rates of CBF among patients with RDP compared to controls.

## 4. Discussion

Our analysis demonstrated differences in CBF in key brain regions between RDP patients when compared to variant-negative controls, with a statistically significant decrease in CBF within the thalamus. There were no corresponding GM volume differences in these patients, which were analyzed with covariates of age and sex (necessary in a small cohort with a range of ages). While a prior study demonstrated neuropathological changes in symptomatic RDP patients, those were brains of elderly patients many years after the onset of symptoms. Moreover, these were compared with age-matched controls with similar comorbidities of vascular and Alzheimer-related changes (23). The results cannot be directly compared, but the absence of GM volume changes in the present study poses questions for future research. This could indicate that clinical symptoms stem from a function-related mechanism early in the disease course, and that the aging process makes the abnormal circuits more vulnerable to neuropathological change. As such, it will be important to determine whether lower rates of CBF among patients with RDP variants without diminished GM volume reflect the course of disease (i.e., whether a reduction of metabolic activity occurs without or preceding neuronal loss). Establishing this will help clarify whether CBF may be a more sensitive biomarker for clinical manifestations of RDP than neuropathological change and whether or not early intervention might ameliorate GM volume loss.

A reduction of CBF being a biomarker for *ATP1A3* disease is plausible for two reasons. First, Na, K-ATPase consumes much of the energy used by the brain (22) and reduction of activity of the enzyme in RDP may reduce this metabolic component directly or magnify it through the potentiation of inhibitory neurons. Second, a reduction in metabolism may result from the perturbation of synaptic activity resulting from the role of Na, K-ATPase in the support of membrane potential, sodium: calcium exchange, and transmitter uptake. A net reduction in neuronal activity is reflected in neuron-glia interactions that result in vasoactivity (34).

The involvement of the thalamus in patients with RDP suggests a potential motor planning and feedback correction mechanism. This is consistent with the type of circuit disorder expected in dystonia and parkinsonism, as well as with the executive dysfunction that we have demonstrated in other patients with RDP (33). We might therefore have expected to observe a relationship between motor function (UPDRS and BFMDRS) and MRI measures. This was not seen in our study. One possibility is that degree of motor dysfunction might be a function of circuit disorganization rather than decreased activity and so may not be reflected in blood flow changes. Another is that detection of a correlation was disfavored by the need for immobilization. Further investigation will be necessary to clarify this issue.

Previous neuroimaging investigations of other genetic dystonias have revealed structural and functional changes in the brain involving components of the CSPTC and CbTC pathways (13–17). In the present study, RDP-associated abnormalities in CBF were identified in the thalamus, which is a component of the CSPTC circuit. These CSPTC regions interconnect with projections from the putamen to the globus pallidus and from the globus pallidus to the thalamus. Our current imaging findings contrast with prior structural and functional neuroimaging studies that have failed to demonstrate RDP-associated abnormalities (6, 8, 35, 36) but are consistent with early SPECT and FDG-PET studies of AHC (10, 12). Conventional diagnostic brain CT and MRI used to evaluate RDP patients have not demonstrated underlying structural abnormalities (35, 36). Functional neuroimaging evaluating the dopamine transporter with ^11^C-2beta-carbomethoxy-3beta-(4-fluorophenyl)-tropane PET and ^123^I-*N*-omega-fluoropropyl-2beta-carbomethoxy-3beta-(4-iodophenyl)-nortropane SPECT as well as evaluating CBF using 99mTc-hexamethylpropyleneamine oxime SPECT have also been unsuccessful in demonstrating RDP-associated abnormalities (6, 8). The absence of abnormalities reported in these previous studies of RDP may reflect limitations in sample size, pathways evaluated, or neuroimaging methods and analysis techniques used at the time the studies were conducted. For example, the previous structural neuroimaging studies did not utilize quantitative voxel-based ROI methods but relied upon less sensitive qualitative visual inspection in the context of clinical diagnostic interpretation of the imaging data. Previous functional neuroimaging PET and SPECT studies were limited in spatial resolution, decreasing the sensitivity of these methods for identifying RDP-associated abnormalities. Furthermore, the PET and SPECT ligand-binding agents are specific for the dopamine transporter, which may be less affected in RDP compared to movement disorders such as idiopathic Parkinson's disease. Finally, it is possible that detection paralleled severity when comparing the early RDP studies with AHC studies. Future studies combining MRI with quantitative molecular imaging (e.g., PET) and radiologic-pathologic correlation will be helpful in this regard. Given the primary finding of RDP-associated changes in brain function (i.e., CBF), additional future directions include evaluation of distributed functional networks using blood oxygen level dependent functional MRI and targeted ROI evaluations of cerebral metabolism using fluorodeoxyglucose (18F) positron emission tomography.

### 4.1. Limitations

There are multiple limitations of our study. Our sample size is small due to the low prevalence of the disease. However, this study remains the largest neuroimaging investigation of symptomatic patients with RDP to date. The findings should be validated in a larger cohort of patients with RDP once that is available. The variability of disease duration in this cohort could have obscured structural abnormalities or metabolic changes, although fixity of symptoms over time is typical for RDP. It is well known high-iron content in regions like the pallidum can reduce the accuracy of segmentation using VBM, thus the lack of between-group structural differences should be interpreted with caution. In addition, ROIs were averaged across hemispheres and were quite large (particularly in the cerebellum), which could have masked subtle between-group structural differences.

### 4.2. Conclusions

Our findings demonstrate reduced CBF in the thalamus among patients with RDP variants compared to healthy variant-negative controls. In addition, detection of between group CBF differences without corresponding GM volume differences supports the hypothesis that RDP may be a disorder of circuit function rather than structural abnormality. If neuronal death/volume loss is not a primary feature of the disease, particularly in relatively younger patients, then therapeutic interventions with functional efficacy could be targeted to prevent the cell/volume loss seen in older RDP patients (23). These findings could identify potential neuroimaging biomarkers that may reflect the underlying processes of RDP and help inform future studies linking genetic susceptibility, environmental triggers, and clinical manifestations of this rare and unique disease.

## Data availability statement

The raw data supporting the conclusions of this article will be made available by the authors, without undue reservation.

## Ethics statement

The studies involving human participants were reviewed and approved by Wake Forest University Ethics Committee. Written informed consent to participate in this study was provided by the participants' legal guardian/next of kin.

## Author contributions

CW, IH, and AB: conception, organization, and execution in research project. CW: design, review, and critique in statistical analysis. CW, BS, and IH: writing of the first draft, review, and critique in manuscript preparation. KA, JC, JK, IH, KS, LO, and AB: review and critique in statistical analysis. KA: writing of final draft, review, and critique in manuscript preparation. BS, JK, KS, and LO: conception in research project. BS: design, execution, review, and critique in statistical analysis. JC: execution in research project. JC, JK, KS, LO, and AB: review and critique in manuscript preparation. All authors contributed to the article and approved the submitted version.
